# Effects of a motivational, individual and locally anchored exercise intervention (MILE) on cardiorespiratory fitness: a community-based randomised controlled trial

**DOI:** 10.1186/s12889-019-6556-0

**Published:** 2019-02-28

**Authors:** Kirstine Høj Obling, Kristian Overgaard, Lise Juul, Helle Terkildsen Maindal

**Affiliations:** 1 Department of Public Health, Section for Health Promotion and Health Services, Aarhus University, Bartholins Allé 2, 8000 Aarhus C, Denmark; 2 Department of Public Health, Section for Sport Science, Aarhus University, Dalgas Avenue 4, 8000 Aarhus C, Denmark; 3 Department of Clinical Medicine, Danish Center for Mindfulness, Aarhus University, Jens Chr. Skous Vej 4, 8000 Aarhus C, Denmark

**Keywords:** Physical activity, Community, Accelerometry, Maximal ergometer bicycle test, Randomised explanatory design

## Abstract

**Background:**

Risk factors for chronic disease, including low cardiorespiratory fitness levels (VO_2max_), are often present in middle-aged populations. We aimed to evaluate the efficacy of a motivational, individual, and locally anchored physical activity intervention on increasing VO_2max_ in 30–49 year-olds with low VO_2max_.

**Methods:**

232 adult volunteers with low VO_2max_ were randomised to intervention (*n* = 115) or routine care (*n* = 117). The intervention included four motivational interviews; six months’ free membership to a local sports club; and a GPS-watch/activity monitor for uploading training data to an online platform/community. Routine care was one or two motivational interviews.

Inclusion criteria were low VO_2max_ based on the cut off levels: ≤ 39 and ≤ 35 ml O_2_/kg/min. For 30–39 and 40–49 year-old men respectively and ≤ 33 and ≤ 31 ml O_2_/kg/min for 30–39 and 40–49 year-old women, respectively. The primary outcome was change in VO_2max_ from baseline to twelve months estimated with a maximal ergometer bicycle test. Secondary outcomes included physical activity, biochemical and anthropometric measures, and health-related quality of life. The primary analyses were based on all available data and sensitivity- and predefined sex analyses were performed. The between-group differences were estimated using independent t-tests and presented with 95% confidence intervals.

**Results:**

No significant between-group differences in primary or secondary outcomes were found at twelve months’ follow-up. The mean VO_2max_ change from baseline in the intervention- and routine care (ml/kg/min) was 3.8 (95% CI: 2.6; 5.0) and 3.4 (95% CI: 1.7; 5.2), respectively. No changes in physical activity were observed. The mean VO_2max_ (ml/kg/min) changes from baseline in the intervention- and routine care group in men were: 5.0 (95% CI: 3.5; 6.4) and 3.5 (95% CI: 1.5; 5.4); and in women: 1.5 (95% CI: -0.1; 3.1) and 3.4 (95% CI: -0.1; 7.8), respectively. Significant differences in VO_2max_ between non-completers (44.2%) and completers were observed, 26.3 (95% CI: 25.1; 27.5) vs 28.2 ml/kg/min (95% CI: 27.1; 29.0; *p* = 0.02). Sensitivity analyses did not change the main result.

**Conclusion:**

Offering a multi-component physical activity intervention to 30–49 year-olds with low levels of VO_2max_ had no effect on the change in VO_2max_ from baseline to twelve months compared with routine care.

**Trial registration:**

ClinicalTrials.gov (no. NCT01801956). Registered 1 March 2013.

## Background

Risk factors for chronic disease, including physical inactivity and low cardiorespiratory fitness levels, are often present in middle-aged populations [1]. While these factors are linked, both physical activity and fitness are also separately and independently associated with diabetes, cardiovascular disease and all-cause mortality [2–4]. Early identification of individuals with these risk factors is important since they represent a group who might benefit from targeted physical activity interventions to increase their fitness and reduce their chronic disease risk.

Previous community-based physical activity interventions among free-living adults have often shown disappointing results and have failed to increase physical activity [5–9]. However, many of these studies are limited by subjective measurement of behaviour and outcomes (such as physical activity and fitness), weak study designs and short follow-up periods [6]. We set out to design and deliver a community-based, multi-component physical activity intervention that addressed these limitations by e.g. including objective measurements of physical activity and fitness.

In the Danish municipality of Randers, where the prevalence of poor cardiorespiratory fitness is 51.7% among men and 31.3% among women [10], the health authorities recently introduced a chronic disease screening and prevention programme called ‘Check Your Health’. This programme offered all citizens (n ≈ 26,000) aged 30 to 49 years a health check, including behavioural and clinical measurements [11]. The programme was designed to promote awareness of and improvement in health behavior, including physical activity.

We developed an intervention to promote physical activity and increase cardiorespiratory fitness among those individuals who were found to have low cardiorespiratory fitness levels in the ‘Check Your Health’ programme. Following a literature review (not published), we identified intervention elements that were previously shown to increase physical activity levels, including local anchoring, self-monitoring and motivational interviewing. A needs assessment among our target group [12] showed that the intervention should be integrated into a broad range of community-based organisations and anchored to local physical activity preferences, such as swimming, running and cycling. We therefore offered participants free membership to a local sports club. There is also good evidence that self-monitoring is a particularly effective behavior change technique [13], while motivational interviewing is associated with increases in physical activity and improvements in physiological and psychological outcomes in other community interventions [14–16]. Researchers have also shown that combining self-monitoring techniques with other techniques such as graded tasks, instructions and prompts are more effective than other behavior change interventions [13, 17]. As such, we offered our participants motivational interviewing and a GPS watch, to enable them to self-monitor and share goals and results with any friends via social media [18–20]. Our intervention was called the ‘Motivational, Individual and Locally anchored Exercise intervention’ (MILE) [12].

Following the development of our intervention, we nested a randomized controlled trial within the ‘Check Your Health’ programme and examined the efficacy of the MILE intervention [12] on objectively measured cardiorespiratory fitness and physical activity among individuals with low cardiorespiratory fitness over one year.

## Methods

### Trial design

We conducted a community-based randomized controlled trial of the MILE intervention based on a previously published protocol [12].

### Participants

Recruitment was via the Danish ‘Check Your Health’ prevention programme [11]. All citizens in Randers municipality aged between 30 and 49 years received an invitation from their general practitioner (GP) to attend a health assessment. Citizens participating in a health check from 1 April 2013 to 1 October 2014 and fulfilling the inclusion criteria were invited by health personnel to join the MILE-study. The primary inclusion criterion was low cardiorespiratory fitness (VO_2max_). This was initially estimated by the submaximal Aastrand-Rhyming test [21] using the following cut off levels: ≤ 39 and ≤ 35 ml O_2_/kg/min for 30–39 and 40–49 year-old men, respectively and ≤ 33 and ≤ 31 ml O_2_/kg/min for 30–39 and 40–49 year-old women, respectively [21]. Those individuals taking part in the study were subsequently measured using a maximal test to establish their VO_2max_ (see below). Participants were also required to have access to the internet at home (due to the use of GPS watches). Exclusion criteria were health problems impeding participation in cardiorespiratory fitness testing, beta-blocker medication, a blood pressure above 160/100 mmHg, pregnancy, alcoholism and a lack of ability to communicate with staff. In total, 232 participants were recruited between 1 April 2013 to 1 October 2014.

### The intervention

The intervention was a six month package based on the three areas identified in the literature and the needs assessment: (i) Joining with a local sports club; (ii) a website and a GPS-watch (self-monitoring) and (iii) motivational interviewing [16].

To integrate physical activity into a broader range of community-based organisations, and thereby anchor the physical activity locally [22], we engaged Randers Gymnastic Club, which is a non-profit local sports club situated in the centre of the City of Randers. It also offers various activities throughout the municipality. In order to promote increased physical activity levels, the participants received a six months free membership to Randers Gymnastic Club (valued at 16 Euro per month per participant). They consequently had free choice and the opportunity to join a wide range of activities promoting moderate and vigorous activities.

To motivate and raise awareness of physical activity the participants were also asked to measure their training activities using a GPS-watch (Garmin Forerunner 210; 134 Euro per watch), which we supplied to all participants. Individuals were encouraged to upload training data from this activity monitor at least once a week to Endomondo.com platform (including distance and heart rate). This is an online sports community based on free real-time GPS-tracking of running, cycling, cross-training etc. It is also possible to upload data on activities that are not captured via the GPS-watch e.g. water-based activities. The website allowed participants to complete a training log, to measure training progression e.g. frequency and intensity, and to compare data with other study participants or friends (via the platform and via facebook), offering the potential to create a virtual community. To keep track of uploaded activities the participants had to join a closed group at Endomondo.com. Participants were allowed to keep the GPS watch if they completed follow-up testing.

Participants were offered four motivational interviews: one at baseline, one after three weeks and one after three and six months. These were delivered by four fitness instructors from Randers Gymnastic Club, who were trained for eight hours in the guiding principles underlying motivational interviews: Asking open questions; Affirming; Reflection; Summarising and providing information and advice with permission. The aim of the motivational interviews was to guide the participants to the kind of physical activity that suited them, and to increase their self-efficacy and self-regulation [23].

The first interview was a 90-min ‘face to face’ interview held at Randers Gymnastic Club including introduction to the membership in Randers Gymnastic Club, the National Health Authority’s advice about moderate and vigorous physical activity [24], the GPS-watch and Endomondo.com. The two ensuing interviews were 15-min telephone interviews, including a status on the uploaded activities, to which the instructors had access, and finally a 30-min ‘face to face’ interview. To ensure quality, a guide based on the principles of the motivational interviewing was provided for each interview, and staff registered each delivered interview. Each instructor was paid a total of 67 Euro per participant.

### Routine care (control group)

Routine care was a one-hour motivational interview delivered by an experienced coach at Randers Health Care Centre followed by a second one-hour interview, if requested. This practice represents the current standard of care offered in the municipality of Randers.

**Fig. 1:**
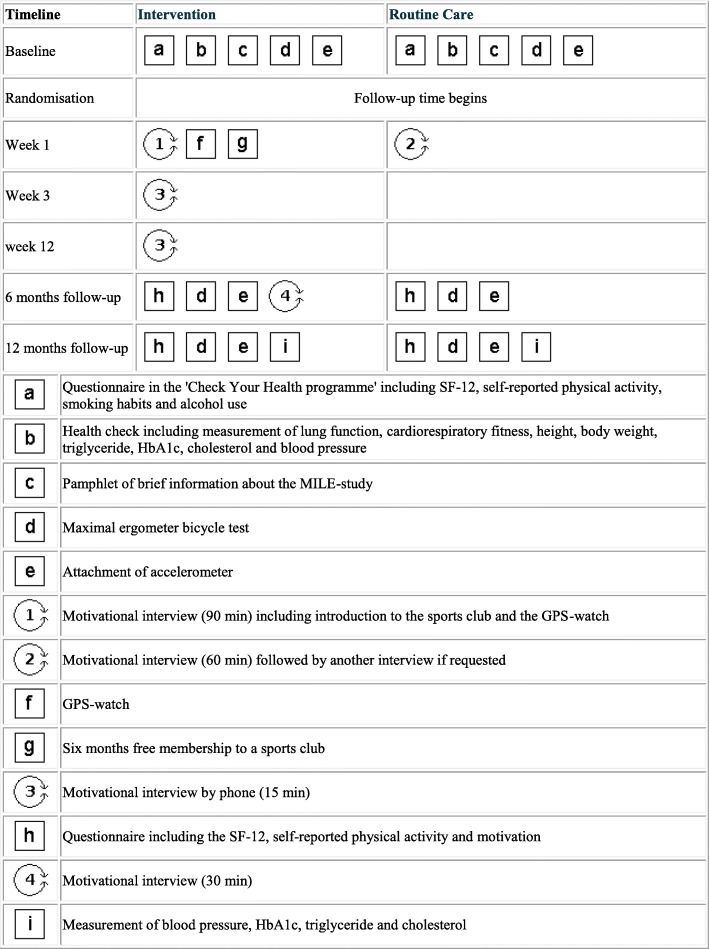
Graphical depiction of the timeline and content of the intervention and routine care in ‘The MILE-study’. Squares reflect the fixed elements, e.g. membership of the sports club. Circles reflect the activities that were flexible, e.g. the motivational interviews. This graphical method was proposed by Perera et al. [25]

The intervention and routine care groups are illustrated using a PaT Plot as proposed by Perera et al. [25], showing the timeline and the characteristics (fixed or flexible) of the components (Fig. 1).

### Outcomes

#### Primary outcome

The primary outcome was a change in VO_2max_ from baseline to twelve months. VO_2max_ is a stable measure that can be measured precisely and can be used as a proxy for habitual moderate to high-intensity physical activity [26]. VO_2max_ wasmeasured using a standardised and validated maximal bicycle ergometer test (Monark 939 E Pendulum Ergometer, Monark Exercise AB, Sweden). The work intensity commenced at 100 watts for men and 75 watts for women. Every second minute the intensity was increased by 35 watt until volitional fatigue. The time spent cycling, the highest intensity achieved and the body weight of the participant were used to estimate VO_2max_ [27]. The primary outcome was measured at baseline, six months and twelve months. For ethical and motivational reasons, the participants were provided with the test results after each visit.

#### Secondary outcomes

At baseline, six months and twelve months we collected objectively measured and self-reported physical activity data; and anthropometric measures. Objectively measured physical activity level was measured using a commercially available accelerometer (Actigraph GT3X-plus) [28]. To avoid non-wear time, the accelerometer was affixed with an adhesive patch on the right hip at the anterior axillary line of the participant, who was required to wear the accelerometer day and night for seven whole days. Participants were also asked to keep a seven-day logbook on their physical activity and sleep. Data from the accelerometers were sampled at 80 Hz sampling rate and downloaded to Actilife (version 6), using intervals of 60 s. A valid wear day was defined as a day from which a full 24-h accelerometer recording was obtained. Data (weekend inclusive) were exported to a custom-made software programme Actianalyzer (Cuno Rasmussen, Department of Public Health – Sport Science, Aarhus University). In this software, sleep time was separated from wake time by a custom-made algorithm [29], which was checked against daily logs of sleep/wake time. Time classified as sleep by the algorithm but marked as wake in the logbook was classified as wake time. Sedentary time was defined as ≤100 counts per minute (cpm) and Freedson adult cut points [30] were used to define light (101–1951 cpm), moderate (1952–5724 cpm) and vigorous activity (5725+ cpm).

Self-reported physical activity was measured via questions from the Danish National Health profile questionnaire [31].

Arterial blood pressure was measured in the sitting position (Omron M6 Blood pressure monitor, Omron Healthcare Europe B.V) after resting for at least five minutes. Blood pressure was used as a screening measure to ensure that people with severe hypertension were excluded from the fitness test. Body weight (wearing light clothes and no shoes), waist circumference and height were measured to the nearest 0.1 kg and 0.5 cm, respectively. Health-related quality of life (mental score only) was measured by the SF-12 questionnaire [32].

At baseline and twelve months we also measured self-reported health (SF-12 questionnaire [16].) and relevant plasma markers (HbA1c, HDL- and LDL-cholesterol, triglyceride) using a capillary test (DCA Vantage Analyzer, Siemens Healthcare, Siemens AG, Germany, Alere Cholestech LDX System, Alere Denmark).

Measurements were undertaken at Randers Health Care Centre by trained staff following standardised procedures from April 2013 to October 2015.

### Sample size

We estimated an expected difference in VO_2max_ of 3 ml/kg/min (SD = 6.0) between intervention and routine care (control) groups at six months. This was based on unpublished data from a different randomised controlled trial of a health screening programme in Denmark showing a difference in VO_2max_ of 2.1 (SD = 6.0) over a five-year period [32], and previous studies comparing 6 and 24 month-intervention effects of a lifestyle programme reporting differences in VO_2max_ of between 3.64 (SD = 3.5) and 1.34 (SD = 3.37) ml/kg/min respectively [33]. With a power of 90% using a two-sided test at the *p* = 0.05 level, it was necessary to recruit at least 85 participants in each group. To accommodate non-independence within couples, we used robust variance estimation to obtain valid uncertainty estimates and an extra 6 participants in each group were added to the sample size. Anticipating a drop-out rate of 30%, a total of 118 participants were required for each group.

### Randomisation

The randomisation sequence was computer-generated by a statistician who was independent of the study. Block randomisation was applied to ensure that couples living together were randomised to the same group. The allocation to either routine care or the intervention took place at the front desk at Randers Health Care Centre. An e-mail was subsequently sent to either the person in charge of routine care or the person in charge of the intervention (project secretary) to let them know which participant had been allocated to which group.

### Blinding

This was an investigator-blinded study. Due to the activities in the two study groups, it was not possible to blind the participants. The outcome measures, data entry and laboratory analysis were conducted by staff blinded to the participants’ study group allocation. However, it was not possible to prevent the participants from talking to the staff that performed the testing.

### Statistical methods

All available data were analysed according to the included subjects’allocation assignment (regardless of a participant’s degree of compliance to the intervention). The intervention was evaluated as a ‘package’, that included all three components. The effects of the intervention were estimated as the differences in the mean changes in the outcomes after six and twelve months between the intervention- and routine care groups. The between-group differences were estimated using independent t-tests and presented with 95% confidence intervals. Furthermore, within-group changes were estimated using paired t-tests and presented with 95% confidence intervals. After randomization, it appeared that baseline characteristics of smoking status and the score for mental health (SF-12) were unequally distributed between the intervention and the routine care groups. We considered these characteristics as potential confounders and adjusted the intervention effect for these characteristics using multivariable regression. We performed a subgroup analysis by sex.

We did not impute missing data in the intention-to-treat analyses because the dataset did not include enough information to perform valid imputed missing data analysis. However, two sensitivity analyses were performed to account for participants who were lost-to-follow up. One sensitivity analysis assumed that VO_2max_ among those lost to follow-up was unchanged from baseline to twelve months, and another sensitivity analysis assumed that those lost to follow-up improved their VO_2max_ corresponding to the mean improvement in the intervention- and routine care group at twelve months.

## Results

### Participant flow

**Fig. 2:**
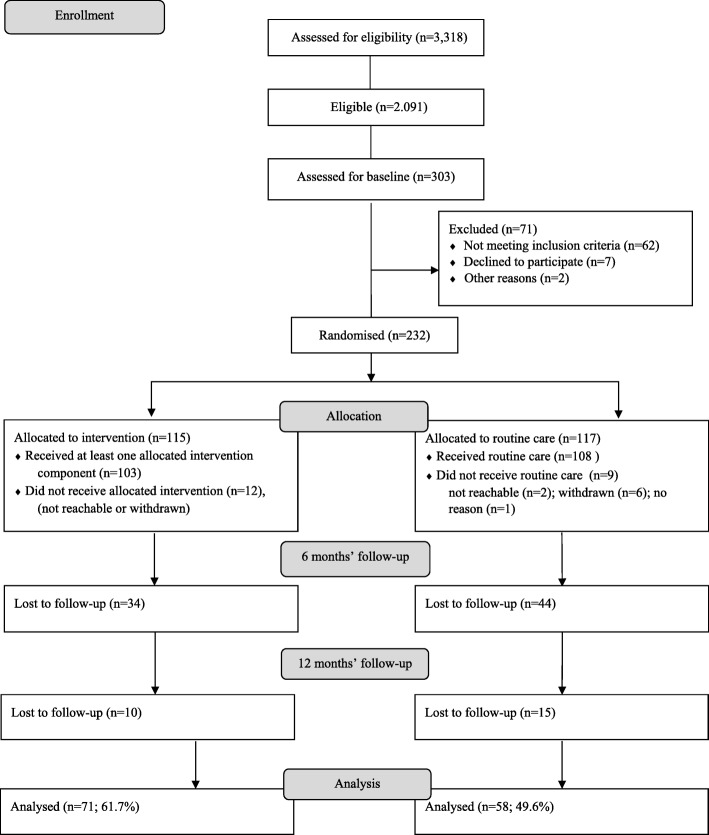
Participant flow in the MILE-study, Randers Municipality, Denmark, 2013–2015

During the study period a total of 3318 participants attended the ‘Check Your Health’ prevention programme in Randers municipality. Of these, 2091 (63%) were classified with low cardiorespiratory fitness according to the Aastrand-Rhyming test. Of these, 303 (16%) participants volunteered for the MILE-study and attended baseline measurement. A further 64 people were excluded at this stage due to hypertension (*n* = 17) and higher fitness levels when tested using the maximal ergometer bicycle test instead of the Aastrand-Rhyming test (*n* = 47). Additionally, seven people chose not to continue in the study after the test. We therefore included 232 participants, 117 in the routine care group and 115 in the intervention group. Due to time constraints, we stopped recruiting when we were four participants short of the required sample size. The flow of the participants is shown in Fig. 2. At twelve months, 44 (38.3%) participants were lost to follow-up in the intervention group, whereas 59 (50.4%) were lost to follow-up in the routine care group.

#### Baseline data

Baseline characteristics of the study population are shown in Table 1. The median age (IQR) was 42.0 years (36.0; 47.0) in the routine care group and 41.0 years (37.0; 46.0) in the intervention group. The majority of the participants were men; 62.4% in the routine care group; and 58.3% in the intervention group. The mean body weight was 2.7 kg more in the intervention group compared to the routine care group, which was also reflected in the waist circumference; 99.0 cm in routine care group; and 100.1 cm in the intervention group. Additionally, the intervention group reported 46.3 min more sedentary time than the routine care group. A total of 20.5% of the participants in the routine care group were smokers compared to 13.9% in the intervention group.

**Table 1:** Baseline characteristics of participants in the MILE-study, Randers Municipality, Denmark, 2013–2015

Characteristics	n	Intervention *n* = 115	Routine care *n* = 117
Sex (male), n (%)	232	67 (58.3)	73 (62.4)
Age (years), median (iqr)	232	41.0 (37.0, 46.0)	42.0 (36.0;47.0)
Weight (kg), mean (SD)	232	89.8 (16.2)	87.1 (16.6)
BMI (kg/m2), mean (SD)	232	29.0 (4.6)	28.2 (4.8)
Waist circumference (cm), mean (SD)	232	100.1 (12.7)	99.0 (14.1)
VO_2max_ (ml/kg/min), mean (SD)	232	27.0 (5.7)	27.4 (5.9)
VO_2max_ (l/min), mean (SD)	232	2.4 (0.6)	2.4 (0.6)
LDL (mmol/l), mean (SD)	219	2.9 (0.7)	2.9 (0.8)
HDL (mmol/l), mean (SD)	229	1.3 (0.3)	1.3 (0.4)
HbA1c (mmol/mol), mean (SD)	231	34.5 (5.2)	34.5 (4.6)
Triglyceride (mmol/l), mean (SD)	231	2.0 (1.1)	2.0 (1.4)
Smoking status (Yes), n (%)	232	16 (13.9)	24 (20.5)
SF-12 mental score, mean (SD)	207	48.2 (10.6)	50.5 (8.6)
Accelerometer measured physical activity, min/day			
Sedentary (0–100 cpm^a^), mean (SD)	223	528.4 (85.8)	530.0 (93.4)
Light (101–1951 cpm), mean (SD)	223	273.8 (69.1)	274.1 (76.7)
Moderate to vigorous^b^ (> 1951 cpm), mean (SD)	223	31.8 (21.3)	34.5 (22.7)
Self-reported physical activity			
Sedentary (min/day), mean (SD)	229	455.6 (232.0)	409.3 (256.2)
Moderate to vigorous > 30 min (days/week), mean (SD)	227	4.0 (2.3)	4.2 (2.1)
Very vigorous > 20 min (days/week), mean (SD)	227	2.2 (1.5)	2.3 (1.6)

^a^cpm = counts per minute

^b^Due to less than two minutes (mean) in vigorous and very vigorous activity at follow-up, these categories were added to the moderate activity category

Both groups had similar levels of VO_2max_, cholesterol, HbA_1c_, triglyceride, objectively measured physical activity and self-reported moderate- and vigorous activity (MVPA).

#### Primary outcome

As shown in Table 2, the intervention group improved their mean VO_2max_ significantly by 2.3 ml/kg/min (95% CI: 1.5; 3.2) at six months and 3.8 ml/kg/min (95% CI: 2.6; 5.0) at twelve months. Likewise, the routine care group improved their mean VO_2max_ significantly at six and twelve months by 1.9 ml/kg/min (95% CI: 1.1; 2.2) and 3.5 ml/kg/min (95% CI: 1.7; 5.2), respectively. The improvements correspond to an increase in VO_2max_ of 14.1% in the intervention group and 12.4% in routine care group from baseline to twelve months’ follow-up. There were no significant differences in the mean change in VO_2max_ in the intervention group versus routine care at twelve months’ (− 0,1; 95% CI: -0.2;0.1). Adjustment for mental health score and smoking status did not change the result.

**Table 2:** Differences in outcome measures between intervention- and routine care groups in the MILE-study at 6 and 12 months’ follow-up

		6 months’ follow-up				12 months’ follow-up		
	n Intervention/Routine care	Intervention Change from baseline, mean (95% CI)	Routine care Change from baseline, mean (95% CI)	∆ between groups (95% CI)	n Intervention/Routine care	Intervention Change from baseline, mean (95% CI)	Routine care Change from baseline, mean (95% CI)	∆ between groups (95% CI)
PRIMARY OUTCOME								
VO_2max_ (ml/kg/min)	80/69	2.3 (1.5;3.2)*	1.9 (1.1;2.7)*	− 0.5 (− 1.6;0.7)	66/47	3.8 (2.6;5.0)*	3.5 (1.7;5.2)*	− 0.3 (− 2.3;1.6)
VO_2max_ (l/min)	80/69	0.2 (0.1;0.3)*	0.1 (0.1;0.2)*	− 0.0 (− 0.1;0.1)	66/47	0.3 (0.2;0.4)*	0.2 (0.1;0.4)*	− 0.1 (− 0.2;0.1)
SECONDARY OUTCOMES								
Weight (kg)	81/73	− 0.9 (− 1.6;-0.2)	− 0.7 (− 1.5;0.2)	0.3 (− 0.8;1.4)	71/58	−1.2 (− 2.3;-0.0)	−0.6 (− 2.3;1.2)	0.6 (− 1.4; 2.6)
BMI (kg/m2)	81/73	−0.3 (− 0.5;-0.1)	−0.2 (− 0.5;0.1)	0.1 (− 0.3;0.4)	71/58	−0.3 (− 0.7;0.0)	−0.1 (− 0.7;0.4)	0.2 (− 0.4;0.8)
Waist circumference (cm)		N/A	N/A	N/A	70/56	−2.7 (−4.3;-1.1)*	− 3.5 (− 5.2;-1.8)*	− 0.8 (− 3.1;1.5)
SF-12 mental score, n (%)		N/A	N/A	N/A	67/49	0.5 (−2.5;3.4)	0.5 (− 1.9;3.0)	0.1 (− 3.9;4.0)
Accelerometer measured physical activity, min/day								
Sedentary (0–100 cpm^a^)	70/61	5.5 (− 12.2;23.1)	−6.7 (− 29.4;16.0)	−12.1 (− 40.2;16.0)	48/35	9.7 (− 13.1;32.4)	6.0 (− 18.4;30.3)	−3.7 (− 37.0; 29.6)
Light (101–1951 cpm)	70/61	−4.4 (− 16.2;7.3)	−4.8 (− 20.9;11.3)	−0.4 (− 19.8;19.0)	48/35	−4.3 (− 21.6;13.0)	−12.3(− 25.6;1.0)	−8.0 (− 30.9;15.0)
Moderate to vigorous (> 1951 cpm)^b^	70/61	1.5 (− 5.8;8.8)	−1.7 (− 6.3;2.9)	−3.2 (− 12.0;5.6)	48/35	−3.9 (− 9.0;1.2)	−6.3 (− 11.1;-1.6)	−2.5 (− 9.6;4.6)
Self-reported physical activity								
Moderate to vigorous > 30 min (days/week)	81/74	−0.8 (−1.2;-0.4)	−0.8 (− 1.3;-0.2)	−0.0 (− 0.7;0.7)	71/56	−0.8 (− 1.4;-0.2)	−0.5 (− 1.1;0.1)	0.3 (− 0.6;1.1)
Very vigorous > 20 min (days/week)	81/74	−0.1 (− 0.5;0.2)	−0.4 (0.9;0.0)	−0.3 (− 0.8;0.3)	71/56	−0.3 (− 0.6;0.1)	−0.4 (− 1.0;0.1)	−0.2 (− 0.8;0.5)
LDL (mmol/l)		N/A	N/A	N/A	63/52	0.2 (− 0.0;0.3)	0.0 (− 0.1;0.2)	− 0.1 (− 0.4;0.1)
HDL (mmol/l)		N/A	N/A	N/A	70/54	0.0 (− 0.0;0.1)	0.0 (− 0.0;0.1)	−0.0 (− 0.1;0.1)
HbA1c (mmol/mol)		N/A	N/A	N/A	70/56	− 0.1 (− 0.5;0.3)	−0.0 (− 0.6;0.5)	0.1 (− 0.6;0.8)
Triglyceride (mmol/l)		N/A	N/A	N/A	70/55	−0.2 (− 0.5;0,2)	−0.2 (− 0.5;0.0)	−0.0 (− 0.5;0.4)

*t-test of significance p ≤ 0.05

^a^counts per minute

^b^Due to less than two minutes (mean) in vigorous and very vigorous activity at follow-up, these categories were added to the moderate activity category

#### Secondary outcomes

As shown in Table 2 both groups had statistically significant reductions in waist circumference from baseline to twelve months. The intervention group reduced their mean waist circumference (cm) significantly by − 2.7 (95% CI: -4.3;-1.1). Likewise, the routine care group reduced their mean waist circumference (cm) by − 3.5 (95% CI: -5.2;-1.8). There were no significant differences in the mean change in waist circumference between the intervention group and routine care groups at twelve months’ (95% CI: -3.1;1.5).

From baseline to twelve months’ follow-up the mean number of complete accelerometer days was 6.7 per week (SD = 0.4) per participant. Aside from a very small reduction in MVPA in the routine care group, there were no other significant changes for any measures over time or between groups.

#### Sex analysis

A total of 80 (57.1%) men and 33 women (35.8%) completed the VO_2max_ test at twelve months’ follow-up. From baseline to twelve months’ follow-up, the men in the intervention- and routine care groups improved their VO_2max_ significantly by 5.0 ml/kg/min (95% CI: 3.5; 6.4) and 3.5 ml/kg/min (95% CI: 1.5; 5.4), respectively. The difference 1.5 ml/kg/min (95% CI: -0.86; 3.86) between groups was not statistically significant. The women in the intervention- and routine care groups showed non-significant changes in VO_2max_; 1.5 ml/kg/min (95% CI: -0.1; 3.1); and 3.4 ml/kg/min (95% CI: -1.0; 7.8), respectively from baseline to follow-up. The between group difference was 2.0 ml/kg/min (95% CI: -1,6;5,5).

#### Characteristics of participants with missing data at 12 months’ follow-up

Due to missing data at twelve months, 44.2% of the study population were considered as lost to follow-up. Significant differences in VO_2max_ at baseline were observed between those lost to follow-up and those completing follow-up; 26.3 ml/kg/min (95% CI = 25.1; 27.5) vs 28.2 ml/kg/min (95% CI = 27.1; 29.0), respectively (*p* = 0.02). In the sensitivity analysis, assuming that VO_2max_ among those lost to follow-up was unchanged from baseline to twelve months, the combined change in VO_2max_ was 2.2 ml/kg/min (95% CI: 1.4; 2.9) in the intervention group and 1.4 ml/kg/min (95% CI: 0.6; 2.1) in the routine care group. In the other sensitivity analysis, assuming that those lost to follow-up improved their VO_2max_ corresponding to the mean improvement in the intervention- and routine groups at twelve months (3.7 ml/kg/min; SD = 5.2), the combined changes in VO_2max_ were 3.7 ml/kg/min (95% CI: 3.1; 4.4) in the intervention group and 3.6 ml/kg/min (95% CI: 2.9; 4.3) in the routine care group.

#### Adherence to intervention

In the intervention group 103 (89.5%) of the 115 participants completed their first motivational interview. The second interview was completed by 87 (75.7%) of the participants, 76 (66.1%) participants completed the third interview and 64 (55.7%) completed the final interview. Data from the sports club show that 71 (62.3%) participants accepted the free membership. Due to resource limitations in the sports club, it was not possible to monitor whether or how often participants engaged in the various activities offered by the club. During the study period 89 (77.4%) participants signed up for Endomondo.com; 16% of these participants did not use the platform.

In the routine care group 108 (92.3%) of the 117 participants took part in the first interview, and 72 (61.5%) participants had a second interview.

## Discussion

### Main findings and comparison with existing literature

We developed an individual- and organizational-based physical activity intervention, which was conducted in a community setting and evaluated in a randomised controlled design. The trial demonstrated that offering a multi-component intervention consisting of motivational interviews, membership to a sports club and a GPS-watch to upload training data to Endomondo.com had no effect on the primary outcome VO_2max_ in 30–49 year-olds compared with routine care. From baseline to six months, both the intervention- and the routine care groups increased their VO_2max_ (ml/kg/min) significantly. This improvement was further increased at twelve months in both groups.

As evidence shows that an improvement in VO_2max_ of 3.5 ml/kg/min in people with low levels of cardiorespiratory fitness is associated with 13 and 15% decreases in risk of all-cause mortality and cardiovascular disease, respectively [3], our intervention was likely to be associated with a significant reduction in risk of all-cause mortality across the whole study cohort. Surprisingly however, no significant increase in objectively measured and self-reported physical activity was seen over the same period of time, except a very small within-group decrease in the routine care group of moderate to vigorous physical activity. This decrease in MVPA was unexpected, and is not in line with the concomitant positive changes in VO_2max_. Therefore, as discussed further below in the limitations section the validity of the accelerometer data may be questioned.

The predefined sub-group analysis of sex showed no significant between group changes in VO_2max_ over time. Worth noticing is that there was a large drop-out especially among women, indicating that the intervention maybe was better suited for men. As discussed later on, this could have been further explored in a process evaluation.

In the literature we identified four randomised multi-component physical activity intervention studies conducted in adults and community settings within the period January 1995 to January 2016 [5, 7–9]. None of these studies had VO_2max_ as outcomes, and comparisons of the results between the different studies were therefore based on physical activity.

The study most comparable to ours was a pragmatic trial by Solomon et al., which provided twelve weeks of locally anchored physical activity for all age groups in 128 rural villages. As in our study, they did not find an effect of the intervention on self-reported physical activity [5], neither did the three other studies [7–9].

Contrary to Solomon et al., we evaluated the efficacy of a multi-component physical activity intervention in a community setting. The purpose of this type of design is to see whether an intervention could work under ideal circumstances. Thus, we used strict inclusion criteria, specialised equipment, closely followed-up participants and measured motivational interview compliance in both participants and practitioners.

While we believed that our locally anchored approach was appropriate in our community setting, there is very little evidence of a positive effect from similar studies. In a review by Baker et al. [34] of 33 community wide interventions for increasing physical activity, very few were effective. The studies were also hampered by serious methodological issues. The overall conclusion from the review is supported by the findings in our study, which circumvented many of the methodological challenges in previous work. In a recent commentary from Millstein et al. [35], the authors bemoan the general lack of coordination in and sustainability of interventions to increase psychical activity. They argue that physical activity research and practice largely remain in siloed fields and disciplines. Future work should therefore focus on local solutions, bringing different disciplines and settings together and to make local structures to serve the broader community. These may vary from improved pedestrian and cycling facilities to general improved infrastructure to support active living [35].

### Strengths and limitations of our study

We used objective measurement of physical activity and fitness. The primary outcome VO_2max_ was measured via an indirect measure, which has good validity (r = 0.88) compared to a gold standard [27]. Physical activity was measured by an accelerometer affixed with an adhesive patch to the right hip day and night for seven days. This method avoids the problem of non-wear time in contrast to most other studies that have used belt-worn accelerometers [36]. However, placing the accelerometer on the hip causes misclassification of certain activities, including cycling, a common mode of transportation and exercise in Denmark. Thus, we may have underestimated levels of MVPA in our study. If cycling activities increased during the intervention, this change would not have been measured in accelerometer recordings, and might partially explain why physical activity did not change from baseline to twelve months but that VO_2max_ did increase. Finally, despite using the recommended ‘best practice’ for accelerometry, which entails measuring physical activity over a period of seven days [37], such measurements may still not reflect the amount of physical activity for the whole intervention period.

It is interesting to note that, despite the low levels of VO_2max_ among our participants, both accelerometer recordings and self-reported physical activity indicated that a significant proportion did fulfill the minimal recommendations for physical activity (30 min daily of moderate to vigorous intensity) at baseline. Although this may seem paradoxical, it is in line with our own observations in a separate study [38], and may indicate that more than 30 min of moderate- to vigorous physical activity per day is required to maintain VO_2max_ in adults of this age group.

It was a strength that the intervention was developed based on learning from previous literature and a local needs assessment. On the other hand, a more detailed intervention development using systematic methods and theoretical frameworks may have improved the intervention as a whole [39].

Another limitation of our study was that we did not have the opportunity or resources to measure the use of activities in the gymnastic club and the frequency, intensity and type of physical activity performed by the participants. The intervention was set up as package, offering participants the option to choose between different intervention components/behavioral techniques, to provide individual motivation and to allow participants to choose the activity that suited them best. However, as we did not monitor the specific techniques used (motivational interviewing and the online platform), we do not have the opportunity to distinguish the effect of one activity from the other. Neither do we have the possibility to access whether the intervention would have had an effect in case of total adherence to all of the intervention components. This limitation could be addressed in future studies. Further, while we chose these intervention components based on a literature review, they were not piloted and may not have been the most appropriate components to encourage increased activity among our target group.

In the study period 2091 people were eligible for participation, 303 (14.5%) of these volunteered, 232 (11.1%) were included and 44.2% of these were lost to follow-up. As those who were lost to follow-up had significantly lower baseline VO_2max_ compared to the completers, and since there was a higher percentage in the control group that were lost to follow-up compared to the intervention group, the potential intervention effect may have been underestimated. This was supported by the results of the sensitivity analysis. One possible reason for higher completion rate in the intervention group may be that the intervention group received a GPS-watch when completing follow-up, which may have encouraged more participants to stay in the study. The direction of this possible bias is, however, unknown. Reasons for the overall large number of participants lost to follow-up may be due to non-compliance with intervention and/or lack of motivation for exercise. This in turn may be linked to the distance to the potential activities in the sports club and/or unwillingness to perform the maximal ergometer bicycle testing, which required a large effort of the participant. By contrast, the close follow-up with repeated testing may also have served as a motivational component for those completing the study since the participants were introduced to the test results after each test. The testing may have been an intervention component in itself causing the improvement in VO_2max_ in both groups (Fig. 1). The effect of measurement has been shown in other physical activity promotion interventions [40]. However, a recent study showed, that including cardiorespiratory fitness assessment in preventive health checks, did not provide higher cardiorespiratory fitness levels at 1-year follow-up compared to preventive health checks without cardiorespiratory fitness assessment [10].

Overall, the interest of engaging in the project was very low (14%), and the dropout rate (44%) was high, indicating a low general acceptance of the intervention and a highly selected study population. It was unexpected that the engagement was overall low, as we had put efforts in a needs assessment. An even more participant-involving planning process may have been advantageously e.g. increased the participant rates. As we did not do that, a process evaluation would have improved the understanding of the trial results. A process evaluation could have investigated the reach of the population and the doses given for each of the intervention components. Further, an analysis of the mechanism that actually produced changes would have supported the understanding of the intervention provided.

This study adds new knowledge on how difficult it is to develop and build interest in a motivational physical activity promotion intervention among a group of middle-aged adults who have many concurrent demands on their time. The development of behavioural interventions that are effective under free living conditions and that effectively reach the intended target population is a complex process requiring systematic research in iterative stages. Onken et al. have proposed the National Institutes of Health (NIH) Stage Model in order to link basic and applied research. The model includes six stages with the two first stages focusing on intervention development. Stage 0 research encompasses basic research about the underlying problems and mechanisms, and stage 1 research includes identifying intervention components, developing and pilot testing complex interventions [41]. We performed a needs assessment and involved members of the target group in the development of the intervention. Based on our findings, we designed a multi-component intervention based on behavioral techniques formerly suggested to be effective. However, the process of development could have been more systematic. For future research, we suggest spending more time on stages 0 and 1 e.g. using co-creation and a theoretical framework in order to develop a sound program theory for increasing participation in physical activity in a way that improves population VO_2max_. We may also have to look beyond individual-level interventions to consider multi-level and population-based approaches, including consideration of the work environment as a health promotion setting [42].

## Conclusions

Offering individuals aged 30 to 49 years with low levels of VO_2max_ a multi-component community wide intervention had no effect on VO_2max_, compared with routine care. The findings must be interpreted by caution, as the study had a poor compliance rate and we were unable to assess which intervention components were taken up by each participant. However, both study groups improved significantly VO_2max_ at six and 12 months. New research should focus on locally adapted programmes using a multi-level approach, with assessment of intervention delivery.

## Abbreviations

CPM: Counts per minute
GP: General practitioner
GPS: Global positioning system
HbA1c: Average blood glucose (sugar) levels for the last two to three months
HDL: High density lipoprotein
kg: Kilogram
LDL: Low density lipoprotein
ml: Milliliter
mmHG: Millimetre of mercury
MVPA: Moderate vigorous physical activity
NIH: National Institutes of Health
O_2_: Oxygen
VO_2max_: The maximum rate of oxygen consumption measured during incremental exercise
